# Choroid plexus enlargement in patients with end-stage renal disease: implications for glymphatic system dysfunction

**DOI:** 10.3389/fneur.2024.1459356

**Published:** 2024-10-14

**Authors:** Sihyung Park, Bong Soo Park, Ho-Joon Lee, Chang Min Heo, Junghae Ko, Dong Ah Lee, Kang Min Park

**Affiliations:** ^1^Department of Internal Medicine, Haeundae Paik Hospital, Inje University College of Medicine, Busan, Republic of Korea; ^2^Department of Radiology, Haeundae Paik Hospital, Inje University College of Medicine, Busan, Republic of Korea; ^3^Department of Neurology, Haeundae Paik Hospital, Inje University College of Medicine, Busan, Republic of Korea

**Keywords:** glymphatic system, choroid plexus, cognition, ESRD, MRI

## Abstract

**Objectives:**

The choroid plexus plays a role in eliminating detrimental metabolites from the brain as an integral component of the glymphatic system. This study aimed to investigate alterations in choroid plexus volume in patients with end-stage renal disease (ESRD) compared with healthy controls.

**Methods:**

We enrolled 40 patients with ESRD and 42 healthy controls. They underwent brain magnetic resonance imaging (MRI), specifically using three dimensional T1-weighted imaging. We analyzed choroid plexus volumes and compared them between patients with ESRD and healthy controls. The diffusion tensor image analysis along the perivascular space (DTI-ALPS) index was calculated. We compared the DTI-ALPS index between the ESRD patients and healthy controls. Additionally, we evaluated the association between choroid plexus volume and neuropsychological tests results in patients with ESRD.

**Results:**

There were significant differences in choroid plexus volumes between patients with ESRD and healthy controls. The choroid plexus volumes in patients with ESRD were higher than those in healthy controls (1.392 vs. 1.138%, *p* < 0.001). The DTI-ALPS index in patients with ESRD was lower than that in healthy controls (1.470 ± 0.239 vs. 1.641 ± 0.266, *p* = 0.005). There were no differences in choroid plexus volumes between patients with ESRD, regardless of the presence of cognitive impairment. However, among the neuropsychological tests, the scores for word-list recognition in verbal memory were negatively correlated with the choroid plexus volume (*r* = −0.428, *p* = 0.006).

**Conclusion:**

We demonstrated a significant enlargement of the choroid plexus volume in patients with ESRD compared to healthy controls. This finding suggests that patients with ESRD have glymphatic system dysfunction, which may be related to cognitive impairment.

## 1 Introduction

The choroid plexus (CP), located in the brain’s ventricles, is essential for producing cerebrospinal fluid (CSF), which provides buoyancy, regulates volume, buffers ions, and removes waste (1). The CP consists of a highly vascularized stroma covered by an epithelial layer, forming a blood-CSF barrier that regulates molecule and ion passage between the blood and central nervous system (2). The CP epithelial cells produce 400–600 mL of CSF daily through a process involving passive fluid ultrafiltration and active ion transport, facilitated by aquaporins.

The CP also supplies essential nutrients like vitamins C, B12, and folate, and secretes growth factors that promote neural stem cell proliferation. It plays a role in clearing harmful compounds from the CSF, maintaining the extracellular environment for optimal brain function (3, 4). The CSF aids in waste removal and biomolecule exchange within the brain, underscoring the importance of the CP-CSF system in central nervous system maintenance (5, 6).

There have been studies showing that various neurological diseases are associated with CP dysfunction. In patients with Alzheimer’s disease (AD), CP enlargement is associated with increased amyloid-beta deposition. A larger CP volume was correlated with worse cognitive function, including memory and frontal/executive domains. CP volume is also linked to a more rapid decline in cognitive function in patients with AD (7). Additionally, AD is one of the diseases with well-known glymphatic system dysfunction.

The glymphatic system acts as a cleaner to remove accumulated waste from the brain, playing a very important role in maintaining brain homeostasis (8, 9). Recently, dysfunction of the glymphatic system has been reported in various neurological diseases such as sleep disorders and epilepsy as well as degenerative brain diseases like AD and Parkinson’s disease (10, 11). The glymphatic system is particularly active during deep sleep, and it has been reported that when sleep is lacking, waste products accumulate in the brain, reducing brain activity and cognitive function (8, 9).

A diffusion tensor image analysis along the perivascular space (DTI-ALPS) index is another advanced, non-invasive measure that assesses glymphatic system function. The DTI-ALPS index evaluates water molecules’ movement along the perivascular space by using diffusion tensor imaging (DTI) to relative measure of diffusivity (12, 13). It has been applied to investigate glymphatic system function in various neurological disorders (12, 14, 15). There have been also reports that DTI-ALPS index in patients with ESRD is lower than that in healthy controls (16).

In addition to this neurodegenerative disease, varying degrees of cognitive impairment remain a major burden in end-stage renal disease (ESRD). The global prevalence of ESRD has increased, and dialysis remains the major treatment option for ESRD (17). Although dialysis helps manage the various metabolic complications related to impaired kidney functions, 85% of the patients with ESRD experience memory loss, language deficits, and difficulty with executive function (18). Cognitive impairment in patients with ESRD is a multifaceted issue with several contributing factors. The exact causes remain unclear, but they likely involve a combination of vascular, metabolic, and inflammatory factors (19, 20). Patients undergoing hemodialysis are especially susceptible to vascular damage, including small-vessel disease and microinfarcts in the brain. These vascular changes can impair blood flow and oxygen delivery to brain regions, affecting cognition (19). Additionally, uremic toxin accumulation and electrolyte imbalances, such as high serum phosphate levels, can contribute to cognitive decline. Chronic inflammation and oxidative stress may directly damage brain cells and impair cognitive function. In addition, dialysis sessions can lead to fluctuations in blood pressure, fluid balance, and electrolyte levels, potentially affecting brain function. Thus, understanding the kidney-brain interaction as a multidisciplinary concern is important. This complicated pathogenic relationship and the exact modulatory mechanisms underlying ESRD and cognitive impairment remain unclear and require further clarification.

We have recently proposed that cognitive impairment in patients with ESRD is related to alterations in the glymphatic system and brain connectivity (16, 21–24). The CP is an important part of the glymphatic system. However, there have been no studies on the CP volume in patients with ESRD. This study aimed to analyze the CP volumes and evaluate their clinical significance. In addition, we evaluated the difference in the DTI-ALPS index between patients with ESRD and healthy controls.

## 2 Methods

### 2.1 Participants

This study was approved by the Institutional Review Board of our hospital. All the participants provided written informed consent. The study was conducted in a single hospital and focused on enrolling 40 neurologically asymptomatic patients with ESRD. Inclusion criteria for these patients were defined as patients with a glomerular filtration rate of <15 mL/min/1.73 m^2^ necessitating renal replacement therapy, undergoing dialysis for more than 3 months, and having no history of neurological or psychiatric disorders at the time of study enrollment. They had no structural lesions on their brain MRIs. Additionally, a control group of 42 healthy participants, matched for age and sex with patients with ESRD, was enrolled. These control participants had no medical, neurological, or psychiatric history. They also did not show any structural lesions on the brain MRI.

### 2.2 MRI acquisition

Both patients with ESRD and healthy controls underwent brain MRI using the same sequence on a 3T-MRI scanner equipped with a 32-channel head coil from Achieva (Philips Healthcare, Best, Netherlands). Three-dimensional T1-weighted images were acquired using a turbo-field echo sequence with the following parameters: inversion time = 1,300 ms, repetition time/echo time = 8.6/3.96 ms, flip angle = 8°, and voxel size of 1 mm^3^ isotropic. The DTI acquisition parameters were as follows: 32 different diffusion directions, repetition time/echo time = 8620/85 ms, flip angle = 90°, slice thickness = 2.25 mm, acquisition matrix = 120 × 120, field of view = 240 × 240 mm^2^, and *b*-value = 1,000 s/mm^2^.

### 2.3 Choroid plexus volume analysis

**Figure 1:**
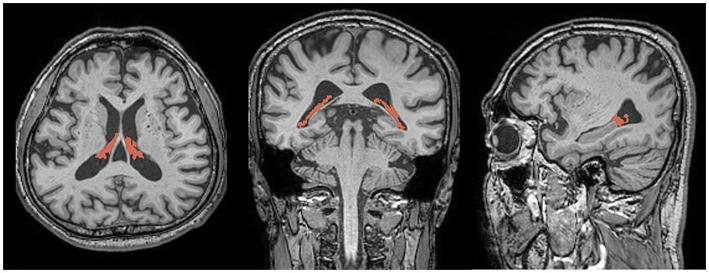
Representative images showing choroid plexus segmentation (red) overlaid on three-dimensional T1-weighted magnetic resonance images in the axial (left), coronal (center), and sagittal (right) planes.

The CP was automatically segmented using Gaussian mixture model-based segmentation, with slight modifications to a previously reported method (25). Briefly, T1-weighted images were corrected for bias field using the Sequence Adaptive Multimodal Segmentation pipeline (26). This correction helps to reduce artifacts caused by variation in the image acquisition process, leading to more accurate subsequent segmentation. Subsequently, the volumes of the right and left lateral ventricle masks and the segmentation-based total intracranial volume were acquired using SynthSeg with the bias-corrected images as input (27). The SynthSeg integrates multiple image modalities to improve the accuracy of the volume measurements. The Gaussian mixture model was then applied to the bias-corrected T1-weighted images to separate the CP from the CSF and ventricular walls as distinct clusters of voxel intensities within the lateral ventricle masks. The Gaussian mixture model assumes that the voxel intensities are drawn from a mixture of Gaussian distributions, which helps in segmentation the CP accurately. A board-certified neuroradiologist with 10 years of experience examined and further refined the CP masks resulting from the automated pipeline to remove any obvious non-CP areas (septum pellucidum, ventricular walls, flow artifacts or noise within the CSF, and so on). The volumes of the final masks (Figure 1) were calculated and normalized using the segmentation-based total intracranial volume.

### 2.4 DTI-ALPS index

The DTI-ALPS index was calculated based on DTI using DSI Studio program. We preprocessed the DTI with following steps: thresholding, smoothing, defragment, and reconstruction. Then, we drew a 5 mm diameter spherical region of interest, in which the lateral projections of the medullary veins were traced orthogonally to the primary diffusion directions in the left hemisphere (12). Next, we obtained the fiber orientation and diffusivities in three directions along the *x*-, *y*-, and *z*-axes as the voxel levels in the region of interest. Among the various voxels, one was selected for each fiber on the same x-axis (projection, association, and subcortical fibers) that presented the maximum orientation in each fiber. The DTI-ALPS index was calculated using the DTI-ALPS index formula: (12) DTI-ALPS index = mean (D*xproj*, D*xassoc*)/mean (D*yproj*, D*zassoc*).

D*xproj*: diffusivity along the *x*-axis in the projection fiber, D*xassoc*: diffusivity along the *x*-axis in the association fiber, D*yproj*: diffusivity along the *y*-axis in the projection fiber, D*zassoc*: Diffusivity along the *z*-axis in the association fiber.

### 2.5 Neuropsychological tests

We evaluated cognitive function in patients with ESRD using a standardized neuropsychological assessment known as the Consortium to Establish a Registry for Alzheimer’s Disease Korean version (CERAD-K). This assessment packet includes evaluations of frontal/executive, language, memory, and visuospatial functions. Scores falling more than −1.5 standard deviations below the mean for age- and education-adjusted norms were considered abnormal. Cognitive impairment was defined when more than one cognitive domain was affected in the CERAD-K.

### 2.6 Statistical analysis

Differences in age, sex, CP volume, and DTI-ALPS index between the groups were compared using an independent *t*-test or Chi-squared test, respectively. Pearson’s correlation test was employed to quantify the associations between the CP volume and neuropsychological tests. Statistical significance was set at a *p*-value of <0.05 for all calculations. All statistical analyses were conducted using MedCalc^®^ Statistical Software version 22.016 (MedCalc Software Ltd., Ostend, Belgium; https://www.medcalc.org; 2023).

## 3 Results

### 3.1 Demographic and clinical characteristics of participants

Table 1 presents the demographic and clinical characteristics of the participants. The age and sex were not different between the patients with ESRD and healthy controls [age, 62.1 ± 6.9 vs. 61.9 ± 7.0 years, *p* = 0.937; male, 19/40 (47.5%) vs. 20/42 (47.6%), *p* = 0.991].

**Table 1:** Demographic and clinical characteristics in patients with ESRD and healthy controls.

	Patients with ESRD (*N* = 40)	Healthy controls (*N* = 42)	*p*-value
Age, years (SD)	62.1 (6.9)	61.9 (7.0)	0.937
Men, *N* (%)	19 (47.5)	19 (47.6)	0.991
Dialysis year, months (SD)	46.5 (52.53)		
Dialysis types (hemodialysis/peritoneal dialysis)	20/20		
Education, years (SD)	10.4 (4.1)		
Hypertension, *N* (%)	40 (100)		
Diabetes mellitus, *N* (%)	17 (42.5)		
Hemoglobin, g/dL (SD)	10.41 (1.17)		
Hematocrit, % (SD)	31.91 (3.68)		
Protein, g/dL (SD)	6.55 (0.63)		
Albumin, g/dL (SD)	3.8 (0.34)		
Aspartate aminotransferase, U/L (SD)	21.1 (6.89)		
Alanine aminotransferase, U/L (SD)	20.13 (10.7)		
BUN, mg/dL (SD)	58.84 (16.72)		
Creatinine, mg/dL (SD)	9 (2.5)		
Sodium, mmol/L (SD)	138.78 (3.25)		
Potassium, mmol/L (SD)	4.81 (0.65)		
Chloride, mmol/L (SD)	99.23 (4.16)		
Calcium, mg/dL (SD)	8.49 (0.68)		
Phosphate, mg/dL (SD)	4.85 (0.94)		
Parathyroid hormone, pg./mL (SD)	286.63 (209.31)		
Total CO_2_ contents, mmol/L (SD)	24.68 (2.62)		

ESRD, end-stage renal disease; SD, standard deviations; BUN, blood urea nitrogen.

Table 2 shows the results of neuropsychological tests in patients with ESRD. Among 40 patients with ESRD, 29 (72.5%) had cognitive impairment. Age and sex did not differ between patients with ESRD, regardless of whether they had cognitive impairment.

**Table 2:** Results of the neuropsychological tests in patients with ESRD.

Neuropsychologic data (*z*-score)	Patients with ESRD (*N* = 40)
**Frontal/executive function**	
Verbal fluency test (SD)	−0.67 (0.94)
Trail making B (SD)	−0.58 (0.94)
Stroop test (reading color words) (SD)	−0.96 (1.14)
**Language**	
Modified Boston Naming (SD)	0.25 (0.86)
**Verbal memory**	
Word list memory (SD)	−0.03 (1.08)
Word list recall (SD)	−0.16 (1.12)
Word list recognition (SD)	0.11 (0.93)
**Visual memory**	
Constructional Recall (SD)	−0.37 (0.95)
**Global cognition**	
MMSE-KC (SD)	0.24 (0.89)
MMSE-KC, raw score (SD)	26.9 (2.8)
MOCA-K, raw score (SD)	22.5 (5.4)

ESRD, end-stage renal disease; BNT-K, Korean version of the Boston Naming Test; MMSE-KC, Mini-Mental State Examination, Korean version of the Consortium to Establish a Registry for Alzheimer’s disease; MoCA-K, the Korean version of Montreal Cognitive Assessment.

### 3.2 Choroid plexus volumes

**Figure 2:**
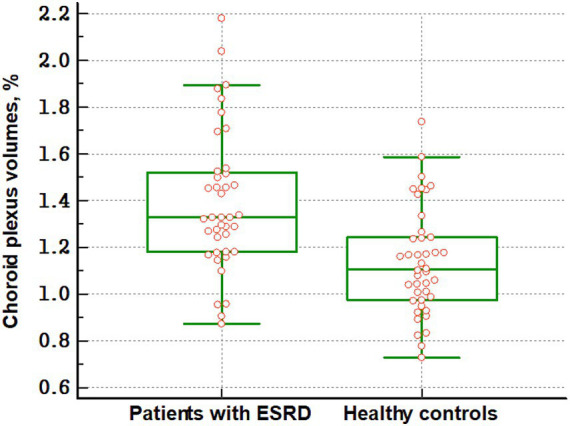
Differences in choroid plexus volumes between patients with ESRD and healthy controls The figure shows that the choroid plexus volumes in patients with ESRD are higher than those in healthy controls (2.514 vs. 2.190%, *p* < 0.001). Increased choroid plexus volumes suggest glymphatic system dysfunction in patients with ESRD.

Figure 2 shows the differences in CP volume between patients with ESRD and healthy controls. There were significant differences in CP volumes between the two groups. The CP volumes in patients with ESRD were higher than those in healthy controls (2.514 vs. 2.190%, *p* < 0.001). However, there were no differences in the CP volumes between patients with ESRD, whether or not they had cognitive impairment (2.506 vs. 2.536%, *p* = 0.837).

### 3.3 DTI-ALP index

The DTI-ALPS index differed significantly between patients with ESRD and healthy controls. The DTI-ALPS index in patients with ESRD was lower than that in healthy controls (1.470 ± 0.239 vs. 1.641 ± 0.266, *p* = 0.005).

### 3.4 Correlation between choroid plexus volumes and age/neuropsychological tests

The scores for the Word-list recognition in verbal memory were negatively correlated with the CP volume in patients with ESRD (*r* = −0.428, *p* = 0.006). However, the other scores in the neuropsychological tests were not correlated with the CP volumes in patients with ESRD, including the Verbal fluency test (*r* = −0.022, *p* = 0.894), Trail-making test type B (*r* = −0.009, *p* = 0.967), Stroop test (*r* = 0.030, *p* = 0.856), Modified BNT-K score (*r* = −0.176, *p* = 0.277), Word-list memory (*r* = −0.083, *p* = 0.610), Word-list recall (*r* = −0.230, *p* = 0.153), Constructional recall (*r* = −0.071, *p* = 0.661), Figure copying (*r* = 0.320, *p* = 0.053), and MMSE-KC score (*r* = −0.002, *p* = 0.988).

## 4 Discussion

In this study, we provided the first report of structural alterations in the CP volume in patients with ESRD. We also confirmed that the DTI-ALPS index was lower in patients with ESRD compared to healthy controls. In addition, word-list recognition in verbal memory was negatively correlated with CP volume in patients with ESRD.

Cognitive impairment in chronic kidney disease begins early and parallels kidney function decline due to endothelial damage (28). Albuminuria and microalbuminuria, markers of renal endothelial damage, can predict cognitive decline. Higher levels of albuminuria are linked to increased cognitive impairment risk (29, 30). As kidney function worsens, uremic toxins such as indoxyl sulfate and para-cresyl sulfate accumulate, posing risks to cognitive function (31). These toxins can damage neurons, disrupt neurotransmitters, and compromise the blood–brain barrier (BBB), which is crucial for protecting the brain from harmful substances in the blood. The BBB’s role in maintaining central nervous system stability highlights its importance in CKD-related cognitive impairment (32, 33).

The blood-CSF barrier, primarily governed by the CP, plays a critical role in pathogenesis and progression of neurological disorders by mediating inflammatory reactions from the body to the central nervous system (CNS) (34, 35). The CP, a highly vascularized structure in the brain’s ventricles, produces CSF through its epithelial cells. Its functions extend beyond CSF production, influencing brain development, neurophysiological processes, and cognitive functions (36). Structural changes in the CP, particularly during aging and in neurodegenerative diseases like AD, affect its integrity and function, potentially contributing to disease progression. In AD, morphological changes in the CP, including epithelial atrophy and basement membrane thickening, are associated with decreased CSF secretion and transport capacity. Studies suggest that CP enlargement in AD patients may be relate to amyloid-beta deposition and impaired clearance, indicating its involvement in disease pathology (7, 37, 38). Similarly, in multiple sclerosis, the CP serves as a gateway for immune cell entry into the brain, highlighting its role in neuroinflammation (39). Furthermore, the CP is implicated in gut microbiota-immune interactions, influencing both gut and brain immune responses. Disruptions in CP integrity, such as volume enlargement, may reflect inflammation and immune-mediated processes rather than disease-specific markers, emphasizing its multifaceted role in CNS health (40).

Abnormalities in the CP function are associated with mood, psychosis, and cognitive dysfunction (41–43). In conditions such as schizophrenia, schizoaffective, and bipolar disorders with psychosis, CP volume enlargement has been observed, along with worse overall cognition, particularly in verbal fluency, attention, and speed of information processing. Studies have linked greater CP volume to smaller gray matter and subcortical volumes, larger ventricular volumes, and reduced white matter microstructure. CP volume enlargement was also linked to impaired word-list recognition in verbal memory, highlighting the CP’s role in cognitive processes (41–43). There have been many studies on changes in CP volume in degenerative brain diseases such as AD and Parkinson’s disease (25, 44). Although the clinical significance of CP volume changes in neurological disorders remains uncertain, a potential link between CP volume alterations and the glymphatic system function has been proposed. The primary function of the CP is to produce the majority of CSF, renewal, and absorption (45). Additionally, the CP plays a key role in mediating brain clearance pathways that help maintain brain homeostasis, suggesting it may be considered part of the glymphatic system (25, 44, 45). Enlargement of the CP may indicate impaired waste clearance, which could negatively impact cognitive function in patients with ESRD. Since CP plays a pivotal role in the production of CSF, the increase in CP volume in patients with glymphatic system dysfunction is thought to be due to the compensatory effect. It is well known that cognitive dysfunction is more common in patients with ESRD than in the general population, likely due to accumulation of waste products in the brain caused by glymphatic system dysfunction. The blood–brain barrier (BBB) is a highly selective, semipermeable barrier composed of capillary endothelial cells, astrocyte end-feet, and pericytes embedded within the basement membrane. It serves to prevent solutes in the bloodstream from crossing indiscriminately into the brain. Additionally, the BBB is part of the glymphatic system, and its dysfunction can contribute to cognitive decline. Various imaging techniques have been developed to study the BBB, including both contrast agent-based and non-contrast agent-based methodologies. There have been reports of BBB disruption in patients with ESRD with function being restored after renal replacement. Furthermore, BBB disruption is associated with cognitive dysfunction (46, 47). Therefore, changes in CP volume, BBB dysfunction, and glymphatic system dysfunction may be interconnected and serve as imaging markers representing cognitive dysfunction in patients with ESRD.

Dysfunction in the glymphatic system, which is responsible for clearing waste from the brain, may be reflected in CP enlargement (48, 49). This suggests a connection between glymphatic system dysfunction and CP enlargement in ESRD, broadening our understanding of cognitive impairment in this context. However, this study has some limitations. First, we enrolled patients with ESRD from a single center, and the sample size of this study was small. The lack of a difference in CP volume between ESRD patients with and without cognitive impairment could also be attributed to the small sample size. Multicenter studies with larger cohorts are required to confirm our findings. Second, because patients with ESRD had underlying conditions such as diabetes mellitus and hypertension, it is difficult to exclude the influence of these diseases on CP volume. However, since diabetes mellitus or hypertension was the most common cause of ESRD, it was challenging to completely eliminate these confounding variables. In the future studies, additional analysis with a control group of patients who have diabetes mellitus or hypertension, but without ESRD, would be beneficial.

## 5 Conclusion

We demonstrated an enlargement of the CP volume in patients with ESRD compared to healthy controls. This finding suggests that patients with ESRD have glymphatic system dysfunction, which may be related to cognitive impairment.

## Data Availability

The original contributions presented in the study are included in the article/supplementary material, further inquiries can be directed to the corresponding author.
